# Characteristics of pleural effusion with a high adenosine deaminase level: a case–control study

**DOI:** 10.1186/s12890-022-02150-4

**Published:** 2022-09-21

**Authors:** Masafumi Shimoda, Aya Hirata, Yoshiaki Tanaka, Kozo Morimoto, Takashi Yoshiyama, Kozo Yoshimori, Takeshi Saraya, Haruyuki Ishii, Ken Ohta

**Affiliations:** 1 Respiratory Disease Center, Fukujuji Hospital, Japan Anti-tuberculosis Association, 3-1-24 Mastuyama, Kiyose City, Tokyo 204-8522 Japan; 2 Department of Respiratory Medicine, Kyorin University School of Medicine, Mitaka City, Tokyo Japan

**Keywords:** Pleural effusion, Adenosine deaminase, Tuberculous pleurisy, Malignant pleural effusion, Pleural infection

## Abstract

**Background:**

Increased pleural fluid adenosine deaminase (ADA) is useful for diagnosing tuberculous pleurisy (TB), but high ADA levels are associated with other diseases. In this study, we compare various disease characteristics in patients with high-ADA pleural effusion.

**Methods:**

We retrospectively collected data for 456 patients with pleural fluid ADA levels of ≥ 40 U/L from January 2012 to October 2021. Cases were classified as TB (n = 203), pleural infection (n = 112), malignant pleural effusion (n = 63), nontuberculous mycobacteria (n = 22), malignant lymphoma (ML) (n = 18), autoimmune diseases (n = 11), and other diseases (n = 27), and data were compared among those diseases. Predictive factors were identified by comparing data for a target disease to those for all other diseases. A diagnostic flowchart for TB was developed based on those factors.

**Results:**

The most frequent disease was TB, though 60.0% of patients were diagnosed with other diseases. Median ADA levels in patients with TB were 83.1 U/L (interquartile range [IQR] 67.2–104.1), higher than those of patients with pleural infection (median 60.9 [IQR 45.3–108.0], *p* = 0.004), malignant pleural effusion (median 54.1 [IQR 44.8–66.7], *p* < 0.001), or autoimmune diseases (median 48.5 [IQR 45.9–58.2], *p* = 0.008), with no significant difference from NTM (*p* = 1.000) or ML (*p* = 1.000). Pleural fluid lactate dehydrogenase (LDH) levels of < 825 IU/L were beneficial for the diagnosis of TB. Neutrophil predominance or cell degeneration, white blood cell count of ≥ 9200/µL or C-reactive protein levels of ≥ 12 mg/dL helped in diagnosing pleural infection. Pleural fluid amylase levels of ≥ 75 U/L and a pleural fluid ADA/total protein (TP) ratio of < 14 helped in diagnosing malignant pleural effusion. High serum LDH and high serum/pleural fluid eosinophils helped in diagnosing ML and autoimmune diseases, respectively. The flowchart was comprised of the following three factors: pleural fluid LDH < 825 IU/L, pleural fluid ADA/TP of < 14, and neutrophil predominance or cell degeneration, which were decided by a decision tree. The diagnostic accuracy rate, sensitivity, and specificity for the diagnosis of TB were 80.9%, 78.8%, and 82.6%, respectively.

**Conclusion:**

Cases involving high pleural fluid ADA levels should be investigated using several factors to distinguish TB from other diseases.

**Supplementary Information:**

The online version contains supplementary material available at 10.1186/s12890-022-02150-4.

## Background

Adenosine deaminase (ADA) is an enzyme produced by lymphocytes [1, 2], and an elevated level of ADA in pleural fluid is a useful marker for the diagnosis of tuberculous pleurisy [3–5]. The most widely accepted cut-off value for ADA in pleural fluid for the diagnosis of tuberculous pleurisy is 40 U/L, with a sensitivity and specificity of 92% and 90%, respectively [6]. Previous reports demonstrated that 3.0–49.0% of patients with ADA levels of ≥ 40 U/L had diseases other than tuberculous pleurisy [6]. However, many diseases other than tuberculosis, such as malignant pleural effusion, empyema and parapneumonic effusion (pleural infection), malignant lymphoma (ML), and autoimmune diseases, sometimes show high ADA levels [3, 4, 7–9]. However, there is no report that compares the characteristics of high-ADA pleural effusion. Furthermore, diagnosis of tuberculous pleurisy is sometimes difficult because acid-fast bacillus tests for pleural fluid have low rates of positivity (smear 6%, polymerase chain reaction [PCR] 51.4%, and culture 36%) [10, 11]. The study demonstrates high ADA levels of pleural effusion by comparing each disease.

## Methods

### Study design and setting

**Fig. 1:**
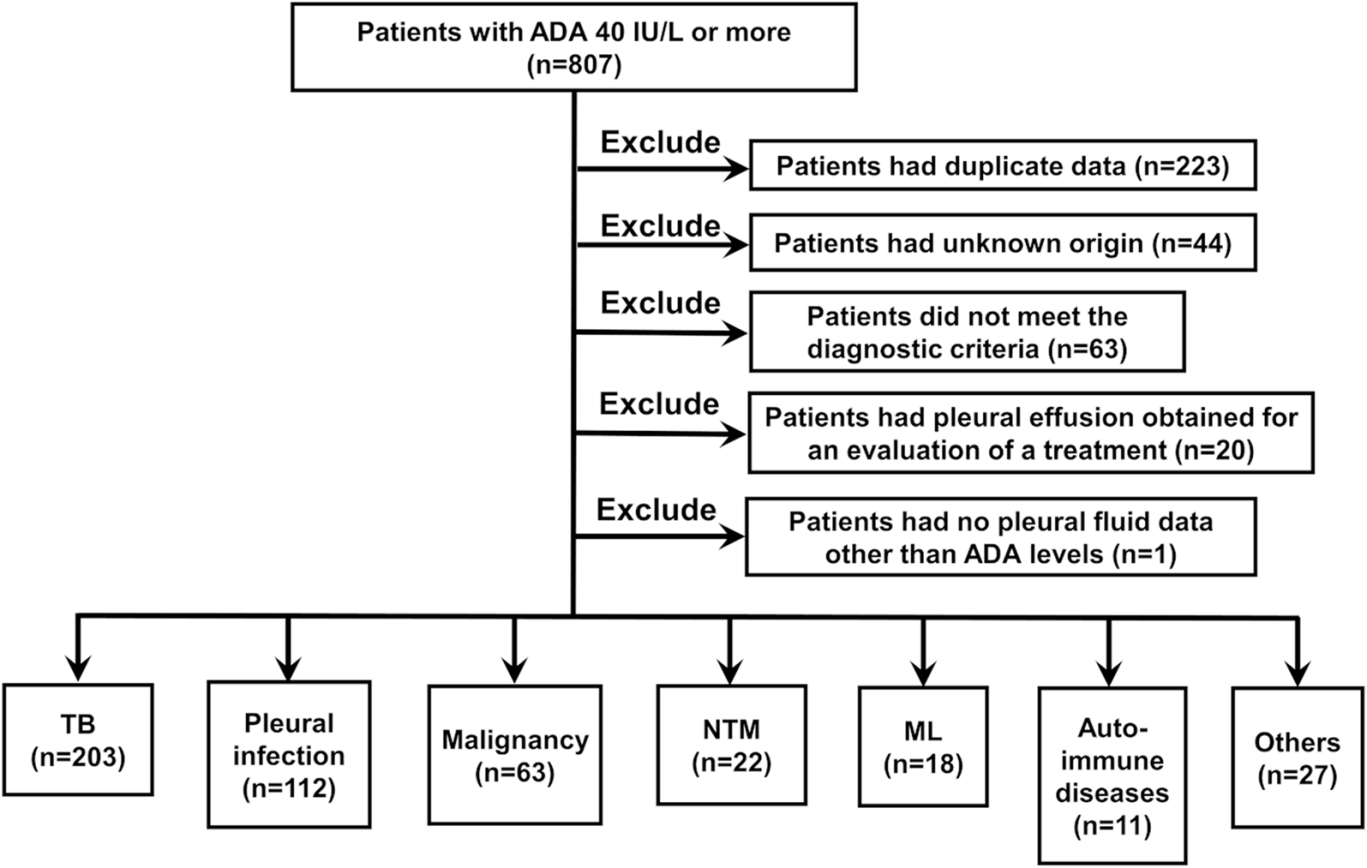
Flowchart of the study. *ADA* adenosine deaminase, *TB* tuberculosis, *NTM* nontuberculous mycobacteria, *ML* malignant lymphoma

We retrospectively collected data from 807 adult patients (age ≥ 18 years) who had high pleural effusion ADA levels of 40 U/L or more at Japan Anti-tuberculosis Association (JATA) Fukujuji Hospital and Kyorin University School of Medicine from January 2012 to October 2021. Excluded patients consist of 224 patients with duplicate data records, 44 with uncertain reasons for pleural effusion, 63 not meeting the diagnostic criteria, 20 with pleural effusion obtained for an evaluation of treatment, and 1 with no pleural fluid data other than ADA levels; ultimately, 456 patients were reviewed. They were classified into 203 patients with tuberculous pleurisy, 112 with pleural infection, 63 with malignant pleural effusion, 22 with nontuberculous mycobacteria (NTM), 18 with ML, 11 with autoimmune diseases, and 27 with other diseases (13 with chronic tuberculous pyothorax, 6 with pneumothorax, 3 with haemothorax, 3 with benign asbestos pleural effusion, and 2 with drug-induced pleural effusion) (Fig. 1), and data were compared among those diseases. Bacteria were identified in specimens from 68 patients with pleural infection, including *Streptococcus* in 38 samples, anaerobic bacteria in 13 samples, *Staphylococcus* in 11 samples, enterobacteria in 2 samples, and others in 8 samples. Malignant pleural effusion was identified in 43 patients with lung carcinoma, 9 with metastatic cancer, and 8 with malignant mesothelioma. NTM results were as follows: *Mycobacterium avium* in 12 patients, *Mycobacterium intracellulare* in 3 patients, *Mycobacterium kansasii* in 2 patients, and *Mycobacterium abscessus* and *Mycobacterium fortuitum* each in one patient. Autoimmune diseases included 5 cases of rheumatoid arthritis (RA), 3 of vasculitis, 2 of immunoglobulin G4-related disease (IgG4-RD), and 1 of Sjögren’s syndrome (SjS). Data regarding symptoms, laboratory test results, radiological findings, and other relevant findings were collected. Furthermore, cut-off values of data for diagnosing a target disease were decided using receiver operating characteristic (ROC) curve analysis, and odds ratios were calculated by binomial logistic regression analysis. Diagnostic, predictive factors were detected by comparing data of a target disease compared to those of all other diseases, and the diagnostic flowchart for tuberculous pleurisy was developed based on those factors. The study was approved by the Institutional Review Board of Fukujuji Hospital (Study number: 21030) and Kyorin University School of Medicine (Study number: R03-216-01), and the requirement for patient consent was waived. All methods were carried out in accordance with relevant guidelines and regulations or the Declaration of Helsinki.

### Definitions

Tuberculous pleurisy was identified in mycobacterial tuberculosis specimens obtained from pleural effusion, pathological findings of caseating granulomatous pleural inflammation without evidence of other granulomatous diseases, or a resolution of pleural effusion after starting anti-tuberculous treatments. Patients with chronic tuberculous pyothorax, a usually asymptomatic disease that occurs as a sequela of collapse therapy for tuberculosis (12), were diagnosed using histologic findings or clinical history and classified into the other group. Pleural infection was identified as bacilli cultured from pleural effusion or a resolution of pleural effusion due to antibacterial treatments. Malignant pleural effusion and malignant lymphoma were diagnosed as malignant cells identified from pleural effusion or a pleural biopsy specimen or as a resolution of pleural effusion after the initiation of anticancer treatment. Pleural effusion due to autoimmune diseases was diagnosed as definitive findings from histological examinations by thoracoscopic pleural biopsy or a resolution of pleural effusion after the initiation of steroid therapy and/or immunosuppressants for diseases diagnosed by each diagnostic criterion [12–15]. No evidence of alternate causes of pleural effusion was included in any of the definitions. Pleural fluid samples from all patients were analysed initially upon diagnosis.

### The evaluation of pleural fluid cell predominance and severe amounts of pleural effusion

Neutrophils were predominantly regarded as having a neutrophil rate of 50% or more in pleural fluid. The cells could not be evaluated in fifty-six samples due to cell degeneration. Severe pleural effusion was defined as a higher-than-normal bronchial bifurcation level on chest radiography [16].

### Statistical methods

Data were analysed and processed using EZR, version 1.53 [17]. The Kruskal–Wallis test and Pearson’s chi-squared test were used for comparing data among 3 groups or more, and Bonferroni’s correction was used for comparative testing. Binomial logistic regression analysis was used for comparing the diagnosis of a target disease to those of all other diseases based on predictive factors and calculated odds ratios. The sensitivity, specificity, and odds ratios were calculated. ROC curves were constructed and used to determine the cut-off values detected by a point of maximum sensitivity and specificity. The level of statistical significance was set at *p* = 0.05 (2-tailed). The diagnostic flowchart for tuberculous pleurisy was developed based on a decision tree, calculated by R (program code was described in the Additional file 1).

## Results

**Fig. 2:**
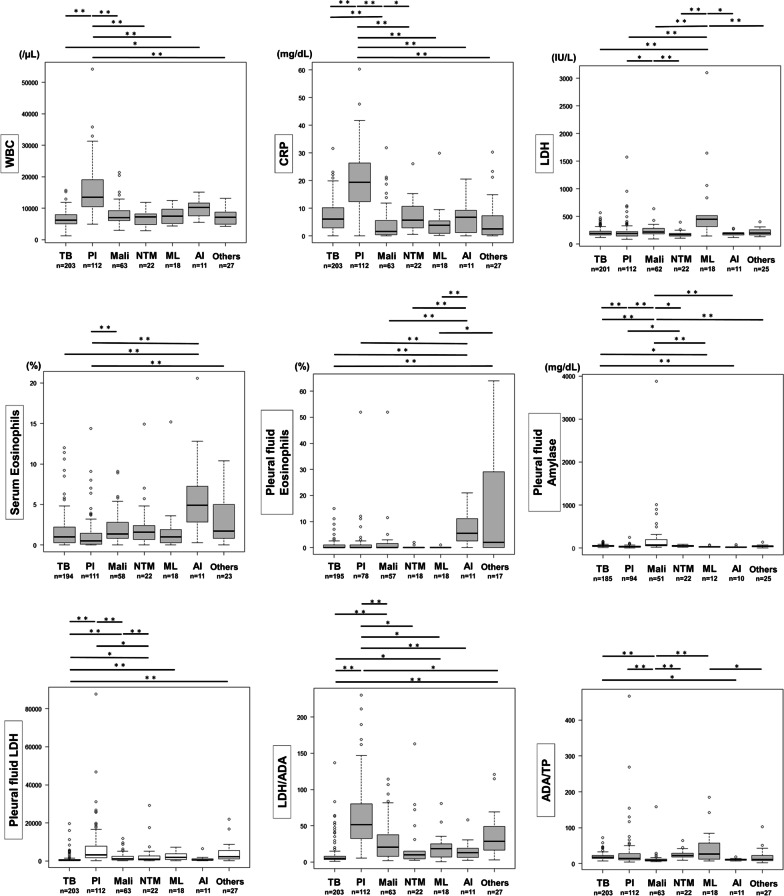
We compared serum and pleural fluid data for 6 diseases by using Bonferroni’s correction. *p < 0.05, **p < 0.001. *TB* tuberculosis, *PI* pleural infection, *Mali* malignancy, *NTM* nontuberculous mycobacteria, *ML* malignant lymphoma, *AI* autoimmune diseases, *WBC* white blood cell count, *CRP* C-reactive protein, *LDH* lactate dehydrogenase, *ADA* adenosine deaminase, *TP* total protein

The rate of patients with tuberculous pleurisy was 40.0% (n = 203), and 60.0% of patients among those with ADA levels of ≥ 40 U/L were diagnosed with other diseases. The baseline characteristics of the study subjects are shown in Table 1, and some factors of serum and pleural fluid were compared among 6 diseases by using Bonferroni’s correction (Fig. 2).

**Table 1:** Baseline characteristics of the study subjects

	TB (n = 203)	Pleural infection (n = 112)	Malignant pleural effusion (n = 63)	NTM (n = 22)	ML (n = 18)	Autoimmune diseases (n = 11)	Others (n = 27)	*p* value*
Age, median (IQR), years	71 (46–83)	69 (60–81)	73 (66–78)	71 (61–79)	81 (74–84)	75 (70–80)	70 (56–78)	0.105
Sex (Male/female)	148/55	97/15	42/21	7/15	11/7	9/2	24/3	< 0.001
Comorbidity, n (%)^a^	143 (71.1)	107 (96.4)	49 (79.0)	18 (81.8)	16 (88.9)	10 (90.9)	27 (100)	< 0.001
Smoking history, n (%)^b^	105 (57.7)	82 (76.6)	43 (69.4)	6 (27.3)	11 (61.1)	7 (70.0)	15 (57.7)	< 0.001
Presence of symptoms, n (%)^c^	178 (90.8)	106 (96.4)	52 (82.5)	21 (95.5)	17 (94.4)	9 (81.8)	21 (77.8)	0.030
Mortality, n (%)	16 (7.9)	7 (6.3)	9 (14.3)	1 (4.5)	5 (27.8)	0 (0.0)	3 (11.1)	0.043
Chest tube insertion, n (%)	37 (18.2)	86 (76.8)	24 (38.1)	16 (72.7)	7 (38.9)	1 (9.1)	14 (51.9)	< 0.001
Laboratory findings								
WBCs, median (IQR), cells/µL^d^	6230 (4990–7920)	13,445 (10,523–18,848)	6960 (6110–9215)	7230 (5183–8080)	7425 (5375–9503)	10,230 (7605–11,640)	7170 (4960–8760)	< 0.001
Eosinophils, median (IQR), %^e^	1.0 (0.3–2.2)	0.5 (0.1–1.5)	1.4 (0.8–2.8)	1.6 (0.7–2.4)	1.0 (0.3–1.9)	4.9 (2.9–7.3)	1.7 (0.8–5.0)	< 0.001
CRP, median (IQR), mg/dL^d^	6.1 (3.0–10.2)	19.4 (12.4–26.3)	1.6 (0.4–5.4)	5.6 (3.1–10.6)	3.9 (1.0–5.4)	6.7 (1.2–9.3)	2.5 (0.5–7.2)	< 0.001
LDH, median (IQR), IU/L ^d^	196 (169–232)	193 (148–229)	222 (189–283)	176 (151–193)	451 (320–514)	191 (167–208)	198 (166–256)	< 0.001
Total protein, median (IQR), mg/dL^d^	6.77 (6.47–7.37)	6.10 (5.53–6.82)	7.13 (6.52–7.64)	6.51 (5.67–7.09)	6.55 (6.06–6.99)	7.22 (6.22–8.13)	7.18 (6.61–7.44)	< 0.001
Pleural effusion								
Neutrophil predominant, n (%)	17 (8.4)	75 (67.0)	8 (12.7)	3 (13.6)	0 (0.0)	2 (18.2)	6 (22.2)	< 0.001
Cell degeneration, n (%)	7 (3.4)	29 (25.9)	6 (9.5)	4 (18.2)	0 (0.0)	0 (0.0)	10 (37.0)	< 0.001
Eosinophils, median (IQR), %^f^	0.0 (0.0–1.0)	0.0 (0.0–1.0)	0.0 (0.0–1.5)	0.0 (0.0–0.0)	0.0 (0.0–0.0)	5.5 (2.5–11.0)	1.0 (0–1.0)	< 0.001
Total protein, median (IQR), mg/dL	4.69 (4.22–5.25)	4.32 (3.70–5.01)	5.43 (5.00–6.25)	4.67 (3.50–5.36)	4.15 (3.91–4.59)	4.82 (4.23–5.84)	5.49 (4.61–7.79)	< 0.001
LDH, median (IQR), IU/L	395 (255–738)	3103.7 (1713.3–7597.3)	1024.0 (454.8–2440.7)	913.6 (441.3–2685.9)	1771.2 (774.3–3576.5)	593.5 (341.5–1121.0)	2156 (1178–5428)	< 0.001
Glucose, median (IQR), mg/dL	91.0 (66.0–408.5)	10.0 (3.0–75.6)	62.0 (9.5–95.0)	68.3 (20.1–96.3)	66.6 (11.1–123.8)	96.0 (52.5–113.9)	34.2 (9.4–81.5)	< 0.001
Amylase, median (IQR), mg/dL^g^	48.5 (37.0–57.1)	29.7 (21.2–72.4)	73.4 (48.9–197.5)	41.4 (34.3–61.4)	28.5 (22.6–36.9)	19.8 (19.0–24.7)	40.9 (25.0–53.2)	< 0.001
ADA, median (IQR), U/L	83.1 (67.2–104.1)	60.9 (45.3–108.0)	54.1 (44.8–66.7)	96.9 (77.3–111.5)	107.4 (58.9–189.9)	48.5 (45.9–58.2)	62.5 (44.1–157.2)	< 0.001
Radiographic								
location (Right/left/bilateral), n	107/67/29	55/44/13	37/25/1	11/11/0	7/8/3	3/5/3	19/8/0	0.024
Cases of severe pleural effusion, n (%)	34 (16.7)	26 (23.2)	19 (30.2)	2 (9.1)	5 (27.8)	1 (9.1)	6 (22.2)	0.223
Pneumothorax, n (%)	6 (3.0)	14 (12.5)	4 (6.3)	13 (59.1)	0 (0.0)	0 (0.0)	9 (33.3)	< 0.001
Calcification on pleural, n (%)	3 (1.5)	2 (1.8)	1 (1.6)	0 (0.0)	1 (5.6)	1 (9.1)	6 (22.2)	< 0.001

Severe pleural effusion was defined as a higher than bronchial bifurcation level on chest radiography

*TB* tuberculosis, *NTM* nontuberculous mycobacteria, *ML* malignant lymphoma, *IQR* interquartile range, *WBCs* white blood cell counts, *CRP* C-reactive protein, *LDH* lactate dehydrogenase, *ADA* adenosine deaminase

*The Kruskal–Wallis test or Pearson’s chi-squared test

^a^TB n = 201, pleural infection n = 111, malignant pleural effusion n = 62, NTM n = 22, ML n = 18, autoimmune n = 11, other diseases n = 27

^b^TB n = 182, pleural infection n = 107, malignant pleural effusion n = 62, NTM n = 22, ML n = 18, autoimmune n = 10, other diseases n = 26

^c^TB n = 196, pleural infection n = 110, malignant pleural effusion n = 63, NTM n = 22, ML n = 18, autoimmune n = 11, other diseases n = 27

^d^TB n = 201, pleural infection n = 111, malignant pleural effusion n = 63, NTM n = 22, ML n = 18, autoimmune n = 11, other diseases n = 25

^e^TB n = 194, pleural infection n = 111, malignant pleural effusion n = 58, NTM n = 22, ML n = 18, autoimmune n = 11, other diseases n = 23

^f^TB n = 195, pleural infection n = 78, malignant pleural effusion n = 57, NTM n = 18, ML n = 18, autoimmune n = 11, other diseases n = 17

^g^TB n = 185, pleural infection n = 94, malignant pleural effusion n = 51, NTM n = 22, ML n = 12, autoimmune n = 10, other diseases n = 25

Median ADA levels in patients with tuberculous pleurisy were 83.1 U/L (interquartile range [IQR] 67.2–104.1), higher than those of patients with pleural infection (median 60.9 [IQR 45.3–108.0], *p* = 0.004), malignant pleural effusion (median 54.1 [IQR 44.8–66.7], *p* < 0.001), or autoimmune diseases (median 48.5 [IQR 45.9–58.2], *p* = 0.008), with no significant difference from NTM (*p* = 1.000) or ML (*p* = 1.000). Pleural fluid lactate dehydrogenase (LDH) was lower in patients with tuberculous pleurisy (median 395 IU/L [IQR 255–738]) than in those with pleural infection (median 3103.7 IU/L [IQR 1713.3–7597.3], *p* < 0.001), malignant pleural effusion (median 1024.0 IU/L [IQR 454.8–2440.7], *p* < 0.001), NTM (median 913.6 IU/L [IQR 441.3–1771.2], *p* = 0.047), ML (median 1771.2 IU/L [IQR 774.3–3576.5], *p <* 0.001), or other diseases (median 2156 IU/L [IQR 1178–5428], *p* < 0.001). The pleural fluid ADA/total protein (TP) ratio was lower in patients with malignant pleural effusion (median 9.89 [IQR 7.95–12.1]) than in those of tuberculous pleurisy (median 17.7 [IQR 14.9–22.4], *p* < 0.001), pleural infection (median 14.5 [IQR 10.3–27.2], *p* < 0.001), NTM (median 23.1 [IQR 19.1–28.6], *p* < 0.001), or ML (median 26.8 [IQR 15.8–53.1], *p* < 0.001). The pleural fluid LDH/ADA ratio of tuberculous pleurisy (median 5.1 [IQR 3.4–8.0]) was lower than those of pleural infection (median 51.5 [IQR 33.2–78.0], *p* < 0.001), malignant pleural effusion (median 20.6 [IQR 8.0–37.8], *p* < 0.001), ML (median 18.4 [IQR 7.3–24.1], *p* = 0.011), and other diseases (median 28.5 [IQR 16.7–49.1], *p* < 0.001). Pleural fluid amylase was higher in patients with malignant pleural effusion (median 73.4 mg/dL [IQR 48.9–197.5]) than in those with tuberculous pleurisy (median 48.5 mg/dL [IQR 37.0–57.1], *p* < 0.001), pleural infection (median 29.7 mg/dL [IQR 21.2–72.4], *p* < 0.001), NTM (median 41.4 mg/dL [IQR 34.3–61.4], *p* = 0.027), ML (median 28.5 [IQR 22.6–36.9], *p =* 0.002), autoimmune diseases (median 19.8 [IQR 19.0–24.7], *p <* 0.001), or other diseases (median 62.5 mg/dL [IQR 44.1–157.2], *p* < 0.001). Patients with pleural infection were more likely to have predominant pleural fluid neutrophils or cell degeneration (n = 104 [92.9%]) than those with tuberculous pleurisy (n = 24 [11.8%], *p* < 0.001), malignant pleural effusion (n = 14 [22.2%], *p* < 0.001), NTM (n = 7 [31.8%], *p* < 0.001), ML (n = 0 [0%], *p* < 0.001), autoimmune diseases (n = 2 [18.2%], *p* < 0.001), or other diseases (n = 16 [59.3%], *p* < 0.001). Pleural fluid eosinophils of patients with autoimmune diseases (median 5.5% [IQR 2.5–11.0]) were higher than those of patients with tuberculous pleurisy (median 0.0% [IQR 0.0–1.0], *p* < 0.001), pleural infection (median 0.0% [IQR 0.0–1.0], *p* = 0.001), malignant pleural effusion (median 0.0% [IQR 0.0–1.5], *p* = 0.008), NTM (median 0.0% [IQR 0.0–0.0], *p* = 0.004), ML (median 0.0% [IQR 0.0–0.0], *p* = 0.001), and other diseases (median 1.0% [IQR 0.0–1.0], *p* = 0.027).

Regarding serum laboratory findings, white blood cell counts (WBCs) (median 13,445/µL [IQR 10,523–18,848]) and C-reactive protein levels (CRP) (median 19.4 mg/dL [IQR 12.4–26.3]) were higher in patients with pleural infection. Patients with ML had higher serum LDH (median 451 IU/L [IQR 320–514]). Serum eosinophils were higher in patients with autoimmune diseases (median 4.9% [IQR 2.9–7.3]). By radiography, pneumothorax was more commonly found with NTM (n = 13 [59.1%]), as 15 of 22 patients with NTM developed cavitary lesion rupture. Six of 15 patients with pleural calcification were diagnosed with chronic tuberculous pyothorax.

**Fig. 3:**
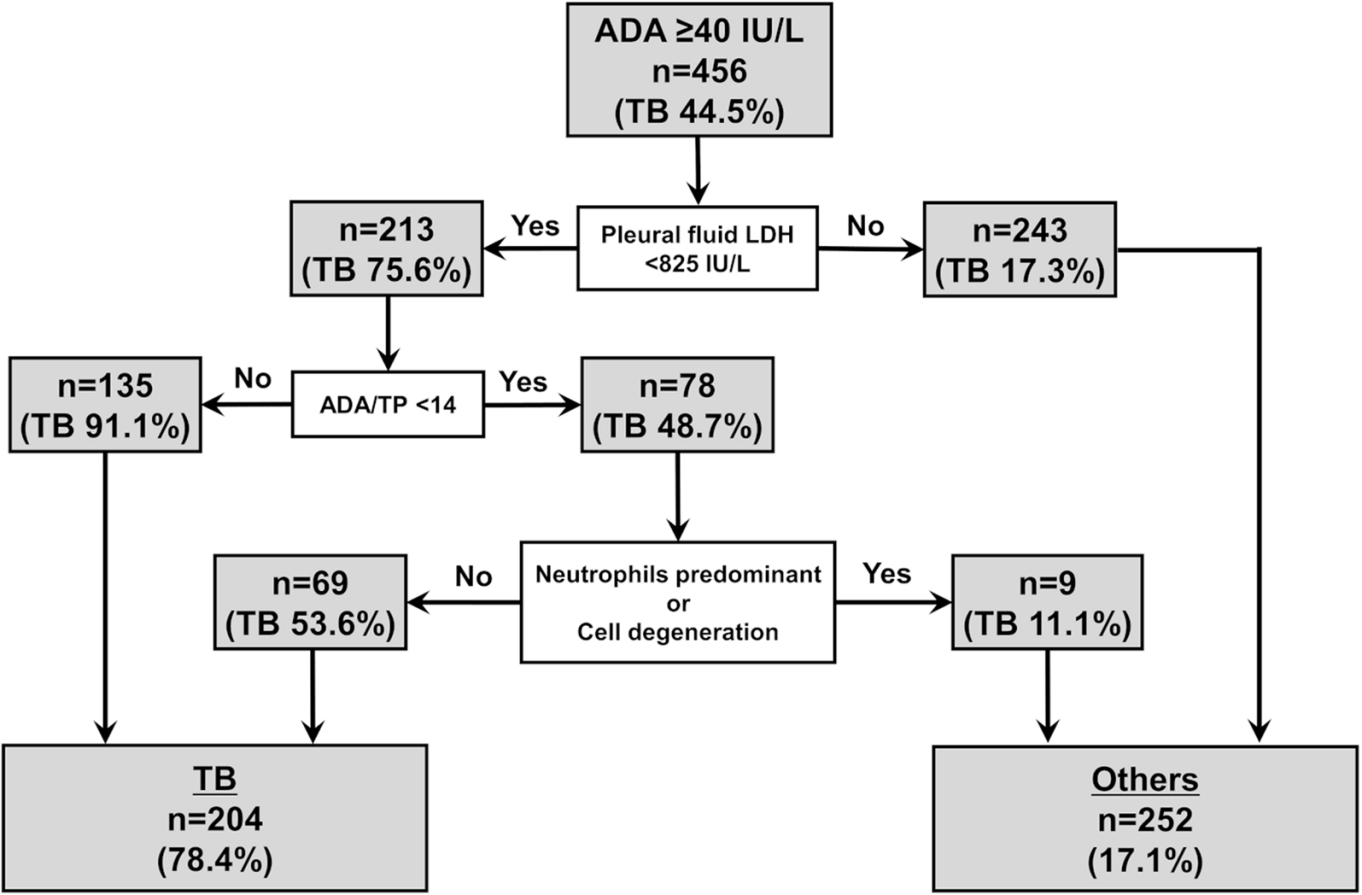
We developed a flowchart for the diagnosis of tuberculous pleurisy. The diagnostic accuracy ratio was 74.3%. *ADA* adenosine deaminase, *TB* tuberculosis, *LDH* lactate dehydrogenase, *TP* total protein

Based on cut-off values, the factors were decided by using ROC (Additional file 2: Table S1). Odds ratios of diagnostic, predictive factors for diagnosing a target disease compared to all other diseases were calculated by binomial logistic regression analysis (Table 2). The odds ratios of pleural fluid LDH < 825 IU/L and LDH/ADA < 26 for diagnosis of tuberculous pleurisy were 12.90 (95% confidence level [Cl] 6.47–25.5) and 4.44 (95%Cl 2.12–9.31), respectively. For diagnosis of pleural infection, odds ratios of neutrophil predominance or cell degeneration and a WBC of ≥ 9200/µL or CRP levels of ≥ 12 mg/dL were 44.60 (95%Cl 19.50–102.00) and 18.60 (95%Cl 8.37–41.1), respectively. Pleural fluid amylase levels of ≥ 75 U/L (13.30 [95%Cl 5.81–30.40]) and pleural fluid ADA/TP of < 14 (17.90 [95%Cl 6.90–46.60]) showed high odds ratios for the diagnosis of malignant pleural effusion. Pneumothorax exhibited high odds ratio for diagnosis of NTM (17.60 [95%Cl 6.99–44.10]) and other diseases (5.79 [95%Cl 2.30–14.60]). Odds ratios of serum LDH ≥ 315 IU/L for diagnosis of tuberculous pleurisy, serum eosinophil counts of ≥ 4.4% and pleural fluid eosinophil counts of ≥ 2.0% for the diagnosis of autoimmune diseases, and pleural calcification for diagnosis of other diseases were 31.70 (95%Cl 10.00–101.00), 58.30 (95%Cl 14.10–241.00), and 16.70 (95%Cl 5.01–56.00). Furthermore, the cut-off value was an ADA of 104.1 IU/L, which is the upper range of ADA in patients with tuberculous pleurisy; because the range of ADA was much higher in patients with ML than those with tuberculous pleurisy, ADA ≥ 104.1 IU/L in patients with ML was more common than in patients with diseases other than ML (n = 9 [50.0%] vs. n = 104 [23.7%], *p* = 0.0216). However, there was no significant difference between tuberculous pleurisy and non-TB cases (TB n = 51 (25.1%) vs. non-TB n = 62 (24.5%), *p* = 0.966) or between tuberculous pleurisy and ML based on Bonferroni’s correction (*p* = 1.000). We developed a flowchart to diagnose tuberculous pleurisy among patients with high pleural fluid ADA levels by using the above factors (Fig. 3). The flowchart was comprised of the following three factors: pleural fluid LDH < 825 IU/L, pleural fluid ADA/TP of < 14, and neutrophil predominance or cell degeneration, which were decided by a decision tree. The diagnostic accuracy rate, sensitivity, and specificity for the diagnosis of tuberculous pleurisy were 80.9%, 78.8%, and 82.9% in the flowchart, respectively.

**Table 2:** Predictive factors for the diagnosis of a target disease compared to all other diseases

Factor	Odds ratio	95% Cl		Sensitivity	Specificity	*p*-*value*
		Upper limit	Lower limit			
*TB*						
Pleural fluid LDH < 825 IU/L	12.90	6.47	25.50	79.3	79.4	< 0.001
Pleural fluid LDH/ADA < 26	4.44	2.12	9.31	93.1	56.1	< 0.001
*Pleural infection*						
Neutrophil predominance or cell degeneration	44.60	19.50	102.00	92.9	81.7	< 0.001
WBC ≥ 9,200/µL or CRP ≥ 12 mg/dL	18.60	8.37	41.10	91.1	72.6	< 0.001
*Malignant pleural effusion*						
Pleural fluid amylase ≥ 75 U/L	13.30	5.81	30.40	49.0	92.2	< 0.001
Pleural fluid ADA/TP < 14	17.90	6.90	46.60	88.9	67.9	< 0.001
*NTM*						
Pneumothorax	17.60	6.99	44.10	59.1	92.4	< 0.001
*ML*						
Serum LDH ≥ 315 IU/L	31.70	10.00	101.00	77.8	90.1	< 0.001
*Autoimmune diseases*						
Serum Eo ≥ 4.4% and Pleural fluid Eo ≥ 2.0%	58.30	14.10	241.00	72.7	95.6	< 0.001
*Other diseases*						
Pleural calcification	16.70	5.01	56.00	22.2	98.1	< 0.001
Pneumothorax	5.79	2.30	14.60	33.3	91.4	< 0.001

*Cl* confidence level, *TB* tuberculosis, *NTM* nontuberculous mycobacteria, *ML* malignant lymphoma, *WBC* white blood cell count, *CRP* C-reactive protein, *LDH* lactate dehydrogenase, *ADA* adenosine deaminase, *TP* total protein, *Eo* eosinophils

## Discussion

We demonstrated many characteristics of diseases with high pleural fluid ADA levels. The most frequent disease with ADA levels of ≥ 40 U/L was tuberculous pleurisy; however, approximately 60% of patients were diagnosed with other diseases. The higher the ADA levels are, the higher the likelihood of tuberculous pleurisy is [18], and an ADA level above 70 U/L is more reliable for diagnosing tuberculous pleurisy [18]. However, in our study, diseases other than tuberculous pleurisy were present in 21.8% of patients with ADA levels of ≥ 70 IU/L. ADA is categorized as ADA-1 and ADA-2 [4, 19, 20]. ADA-2 is mainly activated in tuberculous pleural effusion [4, 19]; conversely, low ADA-2 activities are observed in other diseases, such as neoplastic exudate, empyema, and parapneumonic exudate [4, 19]. The ADA-1/total ADA activity ratio improves performance concerning diagnostic accuracy for tuberculous pleurisy [20]. However, only total ADA can be routinely measured in our clinical practice. Therefore, pleural effusion with high ADA levels should not be diagnosed simply as tuberculous pleurisy. This study reports that many factors for distinguishing diseases. These factors can help diagnose patients with high ADA levels; thus, we developed a diagnostic flowchart for tuberculous pleurisy by using predicted factors with high diagnostic accuracy.

Previous reports showed some predictive factors for distinguishing between tuberculous pleurisy and other diseases regardless of ADA levels. The pleural fluid LDH/ADA ratio for pleural infection and malignant pleural effusion [21–23], pleural amylase for malignant pleural effusion [24], the pleural fluid ADA/TP ratio for IgG4-RD [8] and serum LDH for ML [25] have been reported as useful markers, similar to our results. Pleural fluid amylase levels are helpful for the diagnosis of pancreatic diseases and oesophageal rupture [18, 26], while 10% of malignant pleural effusions show high pleural fluid amylase levels [18]. Pancreatic diseases or oesophageal rupture are very rare [24], and there were no patients with pancreatic diseases or oesophageal rupture in our study. Furthermore, the sensitivity of pleural fluid cytology for malignant pleural effusion is reported to be 46% [27]; hence, high pleural fluid amylase levels may indicate the need for further investigations for cytology or histopathology. A predominance of neutrophils in pleural fluid is usually shown in patients with pleural infection [18, 28, 29], whereas approximately 4.5–17% of tuberculous pleurisy cases are neutrophil predominant despite a usual lymphocytic predominance [28]. In our study, 63 patients without pleural infection (13.8%) showed neutrophil predominance or cell degeneration in pleural fluid. The diagnosis of pleural infection should not be decided by neutrophil predominance or cell degeneration in pleural fluid only. In our study, many patients with autoimmune diseases showed eosinophil elevation in their pleural effusions. Eosinophilic pleural effusion, defined as a pleural effusion of at least 10% eosinophils within a white cell differential count, is commonly caused by trauma, infectious diseases, malignant tumours, and several medications, and autoimmune diseases with eosinophilic pleural effusion are rare [30–32]. Only four patients with autoimmune diseases in our study showed pleural fluid with eosinophil counts of ≥ 10%. Other factors in the pleural fluid, such as interferon-gamma and carcinoembryonic antigen (CEA), are also valuable biomarkers for diagnosing tuberculosis and malignancy, respectively (22,34); however, most patients in our study did not have those levels measured.

The diagnostic flowchart based on those predicted factors is a helpful supplement for distinguishing tuberculous pleurisy from other diseases with high ADA levels. The rate of diagnostic accuracy of the flowchart was 80.9%. However, in particular, malignant pleural effusion or NTM might be challenging to differentiate from tuberculous pleurisy. For pleural effusion due to NTM, there was no helpful marker for distinguishing it from tuberculous pleurisy other than the presence of pneumothorax. Those features might be related to the fact that many patients with NTM were treated, including surgical procedures performed in our hospital. Nevertheless, many patients with NTM are diagnosed due to a cavitary lesion rupture; therefore, evaluating disease progression can be helpful in diagnosis. Moreover, a combination of chest CT findings and pleural fluid cytology help in diagnosing malignant pleural fluid or malignant lymphoma [33, 34]. Clinicians should be aware that patients with high ADA levels in pleural effusion might have a disease other than tuberculous pleurisy. Such cases should be considered using acid-fast bacillus testing of pleural fluid and combined pleural fluid cytology, disease progression, chest computerized tomography findings, and the diagnostic flowchart for tuberculous pleurisy.

This investigation had several limitations. It was conducted retrospectively. The number of cases was small for autoimmune diseases, chronic tuberculous pyothorax, pneumothorax, haemothorax, benign asbestos pleural effusion, and drug-induced pleural effusion, and it might affect the statistical analysis. We collected data for the pleural fluid ADA level, and it was unknown how many patients lacked ADA level data. However, the pleural fluid ADA level is generally investigated routinely regardless of gross appearance in our hospitals; therefore, we believe that there were few biases related to this point.

## Conclusion

This study shows that only 40.0% of patients with ADA levels of ≥ 40 U/L are diagnosed with tuberculous pleurisy and that several biomarkers are helpful for diagnosis. A case involving high pleural fluid ADA levels should be investigated using several factors to distinguish tuberculous pleurisy from other diseases.

## Supplementary Information

**Additional file 1**. Program code for development of a decision tree.

**Additional file 2: Table S1.** The area under the receiver operating characteristic curve of predictive factors for the diagnosis of a target disease compared to all other diseases.

## Data Availability

The datasets generated and/or analysed during the current study are not publicly available due to being written in the Japanese language but are available from the corresponding author on reasonable request after being translated to English.

## Abbreviations

ADA: Adenosine deaminase
ML: Malignant lymphoma
PCR: Polymerase chain reaction
NTM: Nontuberculous mycobacteria
RA: Rheumatoid arthritis
IgG4-RD: Immunoglobulin G4-related disease
SjS: Sjögren’s syndrome
ROC: Receiver operating characteristic
IQR: Interquartile range
LDH: Lactate dehydrogenase
TP: Total protein
WBC: White blood cell count
CRP: C-reactive protein
Cl: Confidence level
CEA: Carcinoembryonic antigen
