# Supplementary material for: Characteristics of pleural effusion with a high adenosine deaminase level: a case–control study
Source: BMC Pulm Med. 2022 Sep 21;22:359. doi: 10.1186/s12890-022-02150-4 (PMC9494830; doi:10.1186/s12890-022-02150-4)
Supplement: Supplementary file 1 — Additional file 1. Program code for development of a decision tree. [file 12890_2022_2150_MOESM1_ESM.docx]

Additional file 1. Program code for development of a decision tree

install.packages("rpart")

install.packages("rpart.plot")

install.packages("partykit")

library(rpart)

library(rpart.plot)

library(partykit)

df <- read.csv("[enter the path to the file]",header = T)

head(df)

tree <- rpart(X0TB ~ . ,data=df, method = "class")

summary(tree)

rpart.plot(tree)

plotcp(tree)

tree.cp <- prune(tree,cp=0.016)

plot(as.party(tree.cp))

rpart.plot(tree.cp)

sum(diag(tab)) / sum(tab)
