# Supplementary material for: Characteristics of pleural effusion with a high adenosine deaminase level: a case–control study
Source: BMC Pulm Med. 2022 Sep 21;22:359. doi: 10.1186/s12890-022-02150-4 (PMC9494830; doi:10.1186/s12890-022-02150-4)
Supplement: Supplementary file 2 — Additional file 2: Table S1. The area under the receiver operating characteristic curve of predictive factors for the diagnosis of a target disease compared to all other diseases. [file 12890_2022_2150_MOESM2_ESM.docx]

Table S1. The area under the receiver operating characteristic curve of predictive factors for the diagnosis of a target disease compared to all other diseases

| Factor | AUC | 95% confidence level | |
| --- | --- | --- | --- |
|  |  | Upper limit | Lower limit |
| Tuberculous pleurisy |  |  |  |
| Pleural fluid LDH | 0.826 | 0.786 | 0.865 |
| Pleural fluid LDH/ADA | 0.882 | 0.850 | 0.915 |
| Pleural infection |  |  |  |
| White blood cell count | 0.903 | 0.869 | 0.937 |
| C-reactive protein | 0.858 | 0.815 | 0.900 |
| Malignant pleural effusion |  |  |  |
| Pleural fluid amylase | 0.775 | 0.699 | 0.852 |
| Pleural fluid ADA/TP | 0.821 | 0.771 | 0.871 |
| Malignant lymphoma |  |  |  |
| Serum LDH | 0.863 | 0.748 | 0.978 |
| Autoimmune diseases |  |  |  |
| Serum eosinophils | 0.814 | 0.665 | 0.963 |
| Pleural fluid eosinophils | 58.30 | 14.10 | 241.00 |

AUC area under the curve, LDH lactate dehydrogenase, ADA adenosine deaminase, TP total protein
